# Mutations in the V1 domain of Thai CRF01-AE viruses that confer sensitivity/resistance to broadly neutralizing antibodies

**DOI:** 10.1186/1742-4690-9-S2-P106

**Published:** 2012-09-13

**Authors:** S O'Rourke, G Tatsuno, B Yu, P Phung, K Mesa, B To, K Limoli, T Wrin

**Affiliations:** 1 Monogram Biosciences, San Francisco, CA, USA; 2 Monogram Biosciences, San Francisco, CA, USA

## Background

Antibodies to the V1/V2 domain of gp120 have recently been identified as a correlate of protection in the RV144 clinical trial. To better understand the specificity of broadly neutralizing antibodies) to the V1/V2 domain of Thai CRF01_AE viruses, we analyzed the specificity of antibodies in HIV+ elite neutralizer (EN) sera by swarm analysis.

## Methods

Swarm analysis makes use of the swarm of closely envelope variants that evolve in each HIV-1 infected individual, as a source of naturally occurring and biologically relevant mutations that confer neutralization sensitivity/resistance. Envelopes from clade B and CRF01_AE viruses were tested for neutralization sensitivity/resistance with sera from ENs infected with clade B and CRF01_AE viruses.

## Results

We found five mutations in the V1 domain that affected neutralization sensitivity/ resistance of CRF01_AE viruses. This differed from clade B viruses in which mutations altering neutralization sensitivity/resistance clustered in the V2 domain. Structural studies have shown that the V1/V2 domain of gp120 consists of a four-stranded β-sheet structure. We found that mutations affecting neutralization sensitivity/resistance in Thai CRF01_AE viruses clustered around the exposed turn at the junction of the A-B strands. In contrast, the mutations that altered neutralization sensitivity/resistance in clade B viruses clustered around exposed turns at the junction of the B-C and the C-D strands.

## Conclusion

The present studies suggest that there is a major difference in the antigenic structure of the V1V2 domain between clade B and CRF01_AE envelope proteins. These results suggest that antibodies to the V1 domain of CRF01_AE envelope proteins should be evaluated as a correlate of protection in the RV144 trial. For this purpose, studies using novel proteins and scaffolds, that replicate the structure of conformation- and glycoform- dependent epitopes in the V1/V2 domain, are under investigation.

